# Early and Polyantigenic CD4 T Cell Responses Correlate with Mild Disease in Acute COVID-19 Donors

**DOI:** 10.3390/ijms23137155

**Published:** 2022-06-28

**Authors:** Alison Tarke, Marina Potesta, Stefania Varchetta, Daniela Fenoglio, Marco Iannetta, Loredana Sarmati, Dalila Mele, Chiara Dentone, Matteo Bassetti, Carla Montesano, Mario U. Mondelli, Gilberto Filaci, Alba Grifoni, Alessandro Sette

**Affiliations:** 1 Center for Infectious Disease and Vaccine Research, La Jolla Institute for Immunology (LJI), La Jolla, CA 92037, USA; atarke@lji.org; 2 Center of Excellence for Biomedical Research (CEBR), Department of Experimental Medicine, University of Genoa, 16132 Genoa, Italy; 3 Department of Biology, University of Rome “Tor Vergata”, 00133 Rome, Italy; marina.pote@gmail.com (M.P.); montesan@uniroma2.it (C.M.); 4 Division of Clinical Immunology and Infectious Diseases, Department of Medicine, Fondazione IRCCS Policlinico San Matteo, 27100 Pavia, Italy; s.varchetta@smatteo.pv.it (S.V.); d.mele@smatteo.pv.it (D.M.); mario.mondelli@unipv.it (M.U.M.); 5 Center of Excellence for Biomedical Research (CEBR), Department of Internal Medicine, University of Genoa, 16132 Genoa, Italy; daniela.fenoglio@unige.it (D.F.); gfilaci@unige.it (G.F.); 6 Bioterapy Unit, IRCCS Ospedale Policlinico San Martino, 16132 Genoa, Italy; 7 Department of System Medicine, University of Rome Tor Vergata, 00133 Rome, Italy; marco.iannetta@uniroma2.it (M.I.); sarmati@med.uniroma2.it (L.S.); 8 Infectious Diseases Unit, Polyclinic San Martino Hospital-IRCCS, 16132 Genoa, Italy; chiaradentone@libero.it (C.D.); matteo.bassetti@unige.it (M.B.); 9 Department of Health Sciences (DISSAL), University of Genoa, 16132 Genoa, Italy; 10 Department of Internal Medicine and Therapeutics, University of Pavia, 27100 Pavia, Italy; 11 Department of Medicine, Division of Infectious Diseases and Global Public Health, University of California, San Diego (UCSD), La Jolla, CA 92037, USA

**Keywords:** COVID-19, acute, T cells, SARS-CoV-2, early, breadth

## Abstract

We assessed SARS-CoV-2-specific CD4+ and CD8+ T cell responses in samples from 89 acute COVID-19 patients, utilizing blood samples collected during the first wave of COVID-19 in Italy. The goal of the study was to examine correlations between SARS-CoV-2-specific T cell responses in the early phase comparing mild, moderate, or severe COVID-19 disease outcomes. T cell responses to the spike (S) and non-S proteins were measured in a combined activation-induced marker (AIM) and intracellular cytokine staining (ICS) assay. Early CD4+ T cell responses to SARS-CoV-2 S correlated with milder disease by both AIM and IFNγ ICS readouts. The correlation of S-specific CD4+ T cell responses with milder disease severity was most striking within the first two weeks of symptom onset compared to later time points. Furthermore, donors with milder disease were associated with polyantigenic CD4+ T cell responses that recognized more prominently non-S proteins in addition to S, while severe acute COVID-19 was characterized by lower magnitudes of CD4+ T cell responses and a narrower repertoire. In conclusion, this study highlights that both the magnitude and breadth of early SARS-CoV-2-specific CD4+ T cell responses correlated with milder disease outcomes in acute COVID-19 patients.

## 1. Introduction

Understanding the quality and characteristics of SARS-CoV-2-specific adaptive responses induced by natural infection and vaccination in the memory phase is important to define correlates of protection from infection, reinfection, and disease [1,2,3,4]. Conversely, the study of SARS-CoV-2-specific adaptive responses induced by natural infection in the acute phase is important to address potential correlates of protection from severe disease and serious outcomes such as hospitalization and fatal disease [5,6].

Some evidence suggests that there is a potential contribution by T cells to the modulation of disease severity in SARS-CoV-2 infection [7,8,9]. If T cells indeed contribute to the modulation of disease severity, this would be consistent with the current observations that neutralizing antibodies notably decrease over time, while T cell activity remains more stable in comparison [10,11]. These changes in adaptive responses likely contribute to the increased rates of infection; however, there is still protection from developing severe disease when a coordinated adaptive immune response occurs [8].

Initial studies on COVID-19 patients have demonstrated the presence of SARS-CoV-2-specific, functional T cells and shown that these T cells are not associated with severe disease or acute respiratory distress syndrome (ARDS) [12]. Additional studies have reported on different aspects of T cell reactivity in acute COVID-19 patients [8,9,13] but were limited by cohort size and the distribution of subjects across the COVID-19 disease severity spectrum. 

These studies have been instrumental in shaping the understanding of T cell responses in COVID-19, but there are knowledge gaps to be addressed by a singular, large cohort representing different disease severities. Our study was designed to investigate the potential role of T cells in modulating disease severity in acute COVID-19 patients utilizing a donor cohort collected during the first wave of COVID-19 in Italy. Our findings further contribute to the understanding of how SARS-CoV-2-specific T cell responses differ between mild and severe disease in early COVID-19. 

## 2. Results

### 2.1. Characteristics of the Cohort of Acute COVID-19 Patients

**Figure 1:**
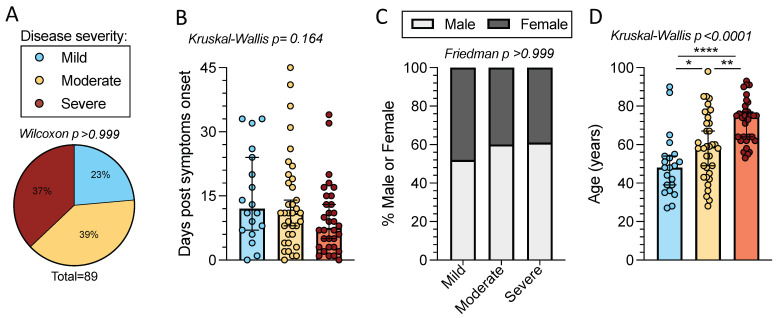
Time from symptom onset, sex, and age distributions of mild, moderate, and severe acute COVID-19 subjects. Peripheral blood mononuclear cells (PBMCs) were collected from 89 acute COVID-19 donors. (**A**) COVID-19 disease severity composition; mild (*n =* 21, blue), moderate (*n* = 35, yellow), and severe (*n* = 33, red). The *p*-value listed above the pie chart was calculated by a one sample Wilcoxon t test. (**B**) Days post-symptom onset (PSO) for the different disease severities. Bars represent the median with 95% confidence intervals. *p*-value is calculated by Kruskal–Wallis t test. (**C**) Percentage of female (dark grey) and male (white) subjects by disease severity. *p*-value is calculated by Friedman one-way ANOVA. (**D**) Age composition for each disease severity category. Bars represent the median and 95% confidence interval and the Kruskal–Wallis t test *p*-value is listed above the graph. When the Kruskal–Wallis t test was significant, Mann–Whitney U tests were applied between disease severities. * *p* < 0.05, ** *p* < 0.01, **** *p* < 0.0001.

In the present study we investigated T cell reactivity with samples from a cohort of acute COVID-19 patients (*n* = 89). Peripheral blood mononuclear cells (PBMCs) from this cohort were collected during the first wave of COVID-19 in Italy, between late February and May of 2020. Due to the limited availability of COVID-19 clinical testing at the time of enrollment, SARS-CoV-2 infection was confirmed in all subjects by either SARS-CoV-2 S RBD PCR and/or antibody tests. All subjects tested for SARS-CoV-2 infection by PCR were positive. Of the subjects who underwent testing for SARS-CoV-2 S antibodies, 96% were positive for IgG (24 of 25 donors) and 81% were positive for IgM (22 of 27 donors). Subjects were relatively well distributed in terms of disease severity (defined as described in the Methods section) with peak disease severities ranging from mild (*n* = 21) to moderate (*n* = 35) to severe (*n* = 33) (*p* >0.999 by one sample Wilcoxon t test) (Figure 1A and Table 1). 

Subjects had blood collected within 0–45 days of symptom onset (overall median of 10 days) at the time of collection. The time from symptom onset was comparable across disease severities (*p* = 0.164 by Kruskal–Wallis t test), with medians of 15, 14, and 10 days post-symptom onset (PSO) for mild, moderate, and severe disease, respectively (Figure 1b). The distribution of sexes was comparable across disease severities; 52% of donors with mild disease were male compared to 60% in moderate and 61% in severe (*p* > 0.999, by the Friedman t test, assuming an even sex ratio of 50% male to 50% female) (Figure 1c). The total cohort spanned a wide age range (27 to 98 years) with an overall median age of 61 years. The median age increased with the severity of disease (median age in years: 48 mild, 59 moderate, and 75 severe), as expected (*p <* 0.0001 by Kruskal–Wallis t test) [8]. Age differences were significant between the disease severity categories (by Mann–Whitney U test: mild–severe *p* < 0.0001, moderate–severe *p* = 0.002, and mild–moderate *p* = 0.027) (Figure 1d) (Table 1). 

### 2.2. CD4 T Cell Reactivity to SARS-CoV-2 Spike

To measure T cell responses to SARS-CoV-2, we utilized a previously described peptide megapool of overlapping 15-mers by 10 amino acids corresponding to the full-length spike (S) protein [14]. We used a combined activation-induced marker and intracellular cytokine staining (AIM + ICS) assay that we previously utilized to measure T cell responses to SARS-CoV-2 [15]. The assay is described in detail in the Methods section and the gating strategy is outlined in Figure S1.

**Figure 2:**
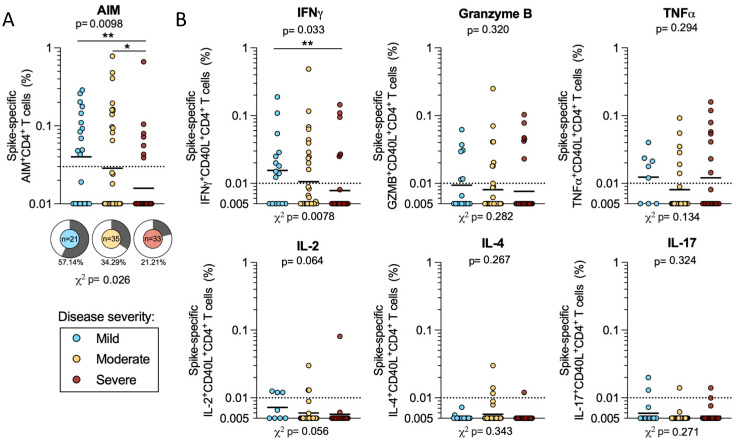
CD4+ T cell responses to SARS-CoV-2 spike in mild, moderate, and severe COVID-19 donors. PBMCs from mild (*n* = 21, blue), moderate (*n* = 35, yellow), and severe (*n* = 33, red) acute COVID-19 donors were tested for CD4+ T cell responses to SARS-CoV-2 S by AIM + ICS assay. (**A**) AIM CD4+ T cell responses were measured by the co-expression of OX40 and CD69. The frequency of response is shown by pie charts. (**B**) The spike-specific cytokine+ CD4+ T cells were measured by the cells expressing CD40L in combination with production of IFNγ, granzyme B, TNFα, IL-2, IL-4, or IL-17. All T cell data shown are background subtracted and SI > 2. Horizontal lines represent the geometric mean and the dotted line represents the limit of sensitivity (LOS), while the y-axis starts at the limit of detection (LOD). *p*-values are calculated by Kruskal–Wallis One-way ANOVA and, when significant, further Mann–Whitney U tests are applied between disease severities. Chi-square test (χ^2^) *p*-values are indicated below the pie charts and are calculated from the frequency of response. * *p* < 0.05, ** *p* < 0.01.

We first considered AIM+ CD4+ T cell reactivity within the cohort of 89 acute COVID-19 donors, as a function of mild, moderate, or severe disease states. Differences in the overall magnitude of spike-specific AIM+ CD4+ T cell responses were significant when all disease severities were considered (*p* = 0.0098 by the Kruskal–Wallis test). The differences between either mild or moderate and severe (geometric means: mild 0.04, moderate 0.029, and severe 0.016) were also significant (mild–severe *p* = 0.002; moderate–severe *p* = 0.039 by the Mann–Whitney U test). Donors with mild COVID-19 also had the highest frequency of responses, with 57% of these donors responding to the spike protein compared to 34% in moderate and 21% in severe. These differences in frequency are also significant (*p* = 0.026 by the Chi-square (χ^2^) test) (Figure 2a). If instead we consider only the COVID-19 subjects with CD4+ T cell responses to the S protein, the magnitude of the response is not significantly different across disease severities (Figure S2a). 

We then quantified the number of CD40L+ CD4+ T cells that were associated with production of several cytokines and secreted factors, as previously described [15]. We found that the majority of the spike-specific responses were associated with the production of IFNγ, Granzyme B, or TNFα. Few donors produced IL-2, IL-4, or IL-17. We observed limited polyfunctionality (data not shown), possibly due to the fact that those responses might develop over time. Among these secreted factors, only IFNγ responses correlated with disease severity and showed significantly higher magnitudes in mild compared to severe COVID-19 (*p* = 0.010). Both the Kruskal–Wallis and Chi-square test were significant (*p* = 0.033 and 0.0078, respectively), indicating that IFNγ responses are significantly higher in milder disease states (Figure 2b). The analysis of only donors with CD4+ T cell responses to the S protein shows similar magnitudes of response for these cytokines and granzyme B (Figure S2b). In conclusion the data support previous reports [8,9] that CD4+ T cell responses are associated with milder disease outcomes.

### 2.3. CD8+ T Cell Reactivity to SARS-CoV-2 Spike

**Figure 3:**
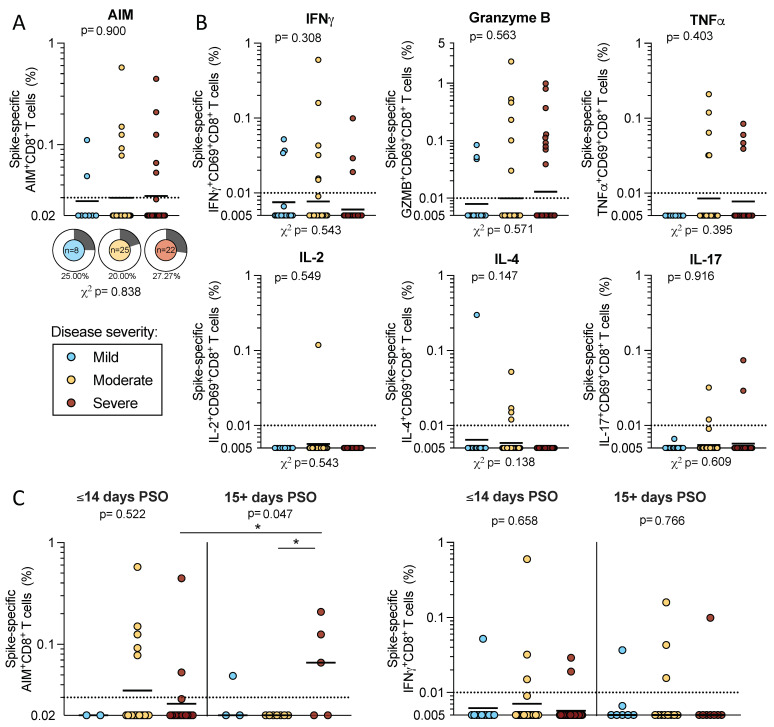
CD8+ T cell responses to SARS-CoV-2 spike in acute COVID-19 donors. PBMCs from acute COVID-19 donors were tested in an AIM + ICS assay for CD8+ responses to SARS-CoV-2 S. (**A**) CD69 + CD137+ AIM + CD8 T cells were quantified in a subset of the total cohort (*n* = 8 mild, 25 moderate, and 22 severe). The frequency of responding donors is indicated by pie charts. (**B**) Spike-specific CD8+ T cell responses to IFNγ, granzyme B, TNFα, IL-2, IL-4, or IL-17 were double positive for CD69. (**C**) AIM+ and IFNγ CD8+ T cell responses were separated by less than 2 weeks or greater than 15 days PSO (3 mild and 1 severe were excluded from the graph because the days PSO was not reported). All data shown is background subtracted and SI > 2. Lines represent the geometric mean and the dotted line represents the limit of sensitivity (LOS), while the y-axis begins at the LOD. *p*-values are calculated by Kruskal–Wallis one-way ANOVA and, when significant, further Mann–Whitney U tests are applied between disease severities and significant results are indicated by the symbols. Chi-square test (χ^2^) *p*-values indicated below the pie charts are calculated from the frequency of response. * *p* < 0.05.

CD8+ T cell responses were also measured by AIM + ICS assay. We first examined AIM+ CD8+ T cell reactivity in a subcohort of 55 acute donors (*n* = 8 mild, *n* = 35 moderate, and *n* = 22 severe) due to the limitations in cell numbers. We observed similar magnitudes of AIM+ CD8+ T cell responses to SARS-CoV-2 spike proteins across disease severities (geometric means: mild 0.028, moderate 0.030, and severe 0.031) that did not differ significantly (*p* = 0.900 by Kruskal–Wallis *t* test). The frequencies of donors with spike-specific AIM+ CD8+ T cells ranged from 20–27% (mild 25%, moderate 20%, and severe 27%), and these differences were not significant (*p* = 0.838 by χ^2^ test) (Figure 3A). 

We also measured spike-specific CD8+ T cells that expressed CD69 in combination with the production of one of several cytokines or granzyme B by ICS in the full cohort of 89 acute donors. As we observed for CD4+ T cells, IFNγ, granzyme B, or TNFα production contributed to the majority of spike-specific responses by CD8+ T cells; few donors had spike-specific CD8+ T cell responses associated with IL-2, IL-4, or IL-17 production. For each of these secreted factors, neither the magnitude nor the frequency of response was significantly different by the Kruskal–Wallis or χ^2^ t tests (Figure 3b). In conclusion, the data show that by both AIM and ICS, CD8+ T cell responses to SARS-CoV-2 are less frequent and more difficult to detect in the early stages of acute COVID-19, and that no significant difference is detected in these responses as a function of disease severity. 

### 2.4. T Cell Reactivity to SARS-CoV-2 Spike in Early and Late Infection 

**Figure 4:**
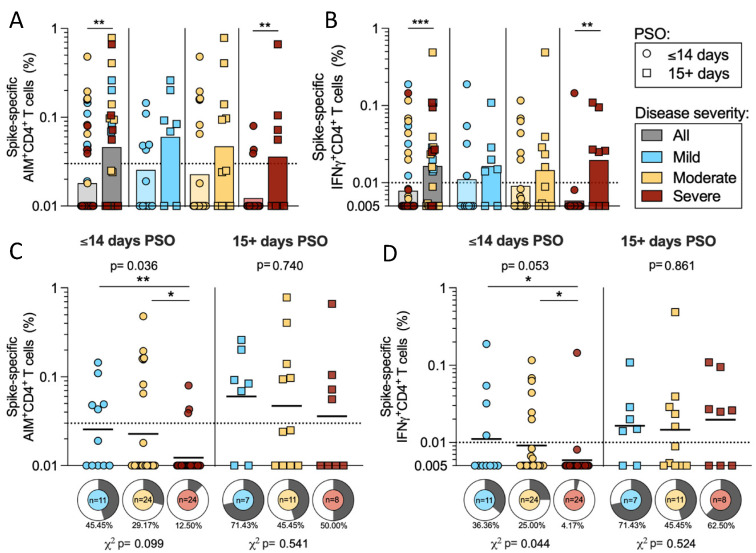
Early and late CD4+ T cell responses to SARS-CoV-2 S. The cohort of acute donors was divided by ≤14 days PSO (early) and 15+ days PSO (late), and the PBMCs were tested for CD4+ T cell reactivity to SARS-CoV-2 spike by AIM and IFNγ ICS. The magnitude of spike-specific CD4 reactivity is shown for mild (*n* = 18, blue), moderate (*n* = 35, yellow), severe (*n* = 32, red), or all (*n* = 85, grey) COVID-19 donors (3 mild and 1 severe were excluded from the graph because the days PSO was not reported). The data are shown for the entire cohort and separated for side-by-side comparisons of early and late PSO within disease severities in AIM (**A**) and IFNγ (**B**) ICS. The same data are plotted as shown to compare the T cell responses in AIM (**C**) and IFNγ (**D**) ICS within disease severities. Additionally, pie charts show the frequency of the response for each level of disease severity. The y-axis starts at the LOD and the dotted line represents the LOS, while the solid lines or bars represent geometric mean. *p*-values are calculated by Kruskal–Wallis across all disease severities and Mann–Whitney U tests are applied as indicated by the symbols when significant. Chi-squared test is applied to the frequencies of response as indicated by the *p*-values below the pie charts. * *p* < 0.05, ** *p* < 0.01, *** *p* < 0.001.

In the next series of analyses, we analyzed responses as a function of time PSO. We focused on the CD4+ T cell responses since by both AIM and ICS assays CD4+ (but not CD8+) T cell responses correlated with disease severity. When we divided the cohort based on the start of symptoms into early (≤14 days PSO) versus later (15+ days PSO) subgroups, responses increased with time by both AIM (*p* = 0.0017) and IFNγ (*p* = 0.0007) (Figure 4a–b). Particularly in severe donors, spike-specific AIM+ CD4+ T cells increased in magnitude from 0.012 geometric mean to 0.036 in the early compared to later timepoints (*p* = 0.0096) (Figure 4a). A similar pattern was seen in the IFNγ CD4+ response, in which the magnitude increased from a geometric mean of 0.006 to 0.02 in severe disease (*p* = 0.0028) (Figure 4b).

Plotting the same data in a different way emphasizes that differences in the magnitude of spike-specific AIM+ CD4+ T cells as a function of disease severity, clearly tracked to the early PSO subgroup (Mann–Whitney U tests: mild–severe *p* = 0.005; moderate–severe *p* = 0.045; Kruskal–Wallis *p* = 0.036). There was also a trend in the differences in the frequency of the responses that were evident in the early PSO donors (*p* = 0.099 by χ^2^ t test). Strikingly, the subgroup of samples collected 2 weeks or further from the start of symptoms was not associated with significant differences as a function of disease severity (Figure 4c).

A similar pattern was seen in the CD4+ IFNγ response, which is shown in Figure 2 to vary as a function of disease severity in the overall cohort. Spike-specific IFNγ+ CD4+ T cell responses were higher in magnitude for both mild and moderate disease compared to severe disease in donors in the early PSO subgroup (*p* = 0.029 and 0.027 by Mann–Whitney, respectively). The frequency of donors responding was also greater in mild and moderate categories compared to severe (χ^2^ *t* test *p* = 0.043) in the early PSO group. In samples collected 2 weeks after the start of symptoms, the spike-specific IFNγ responses in CD4+ T cells were not significantly different across the different disease severities (Figure 4d).

Our analysis did not demonstrate differences in CD8+ T cell reactivity as a function of disease severity in the full cohort (Figure 3). Here, we detected lower spike-specific AIM+ CD8+ T cells in the early severe disease group (geomean 0.026) compared to the late severe disease group (geomean 0.59; *p* = 0.04 by the Mann–Whitney U test). However, within the early and later PSO groups, no differences were observed in the CD8+ T cell responses between disease severities by either AIM or IFNγ ICS (Figure 3c). 

In conclusion, the correlation between CD4+ T cell responses and milder COVID-19 clearly tracks to early time points. Early CD4+ responses are associated with milder disease and are more likely to be absent or impaired in severe COVID-19. 

### 2.5. Sex and Age in Relation to CD4+ T Cell Responses in Acute COVID-19 Donors

**Figure 5:**
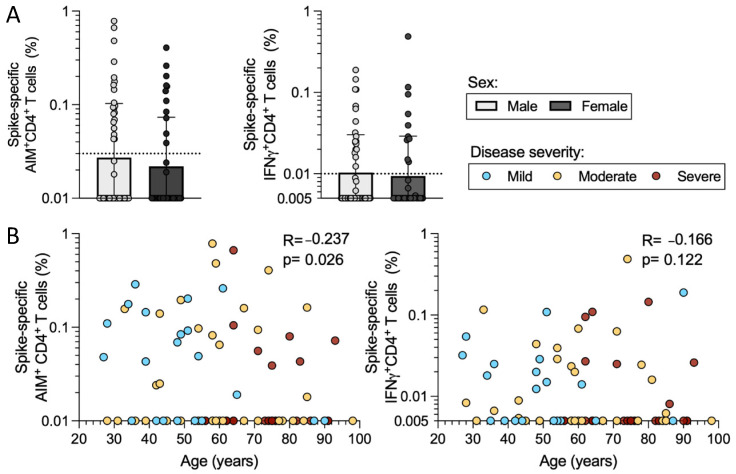
CD4+ T cell reactivity as a function of donor sex and age. The cohort of 89 acute COVID-19 donors was analyzed for CD4+ T cell responses to SARS-CoV-2 S by AIM + ICS assays. (**A**) The spike-specific AIM+ and IFNγ+ CD4+ T cell responses are shown for the entire cohort as a function of donor sex (male in white and female in dark grey). The y-axis begins at the LOD for each assay and the dotted line represents the LOS. Mann–Whitney U tests were applied to the data and the results are indicated by symbols when significant. (**B**) The CD4+ AIM and IFNγ reactivity to S is shown as a function of donor age. Mild (*n* = 21, blue), moderate (*n* = 35, yellow), and severe (*n* = 33, red) disease states are plotted together. R and *p*-values are calculated by Spearman correlation.

SARS-CoV-2 T cell reactivity is influenced by factors such as sex and age [16,17]. As mentioned above, the mild, moderate, and severe disease categories had similar representations of male and female sexes (Figure 1). Here, we examined T cell reactivity as a function of the disease severity. The magnitude of the response in spike-specific AIM+ CD4+ T cell responses was not significantly different in males compared to females, although there was a trend for a lower median response in females. Similarly, the frequency of spike-specific IFNγ CD4+ T cells was comparable in males and females (Figure 5a). 

As mentioned above, the mild, moderate, and severe disease categories in our cohort did significantly differ in terms of age (Figure 1d), with increasing age representation in the more severe disease subgroup. When we plotted the magnitude of AIM+ CD4+ T cell responses as a function of age, we observed a negative correlation with age (*p* = 0.026); however, the extent of the correlation was limited (r = −0.236) and not significant when IFNγ+ CD4+ T cells were considered (*p* = 0.112) (Figure 5b). Taken together, these results suggest that differences in terms of sex and age do not account for the observed differences in T cell reactivity as a function of disease severity.

### 2.6. CD4 T Cell Reactivity to Spike and Non-Spike Antigens

**Figure 6:**
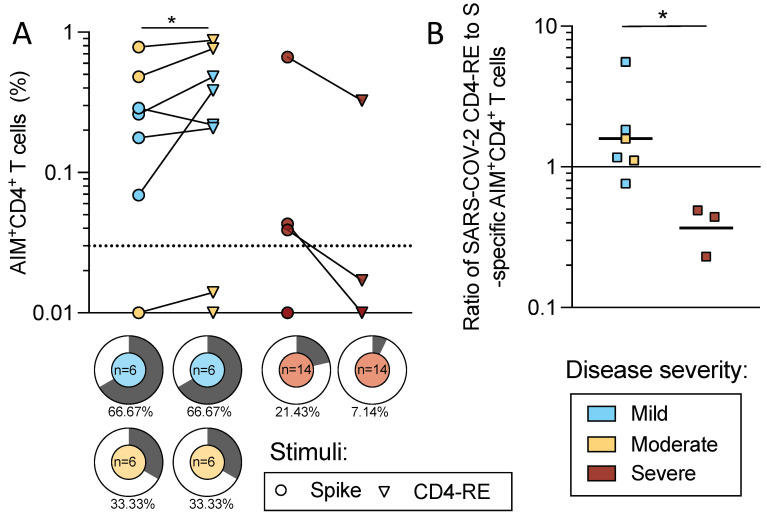
SARS-CoV-2 spike protein and CD4-RE-specific CD4+ T cell reactivity. Mild (*n* = 6, blue), moderate (*n* = 6, yellow), and severe (*n* = 14, red) acute COVID-19 donors were assessed for T cell responses to SARS-CoV-2 S and non-Spike proteins. PBMCs from these donors were assayed with the S peptide pool of overlapping 15-mers by 10 and the CD4-RE pool of experimentally defined non-spike epitopes (Yu et al., Cell Host & Microbe 2022). (**A**) The magnitude for AIM+ CD4+ T cells specific for spike (circles) and CD4-RE (triangles) is shown for mild/moderate and severe COVID-19 donors. The dotted line indicates the LOS for the AIM assay, while the y-axis starts at the LOD. The frequency of positivity is indicated in pie charts for S and CD4-RE for each disease severity. (**B**) The ratios of CD4-RE to S AIM+ CD4+ responses are shown for responding donors (mild *n* = 4, moderate *n* = 2, and severe *n* = 3). Mann–Whitney or Wilcoxon tests were applied and *p*-values are shown for significant values. * *p* < 0.05.

While the results above characterized T cell reactivity to the spike protein, the reactivity to the rest of the genome is also of interest, given that reactivity to the SARS-CoV-2 nucleocapsid (N) and non-structural proteins (NSPs) may potentially contribute to the modulation of disease severity [18,19,20]. The number of PBMC available for analysis was limited, but 26 subjects had a sufficient number of cells available to perform additional analyses related to this issue. To examine T cell responses to the rest of the SARS-CoV-2 proteome, we utilized the approach recently described by Yu et al., based on CD4-RE, a pool of experimentally defined 15-mer epitopes derived from the whole SARS-CoV-2 proteome (excluding S) [21]. The CD4-RE epitope pool was tested in parallel with the S pool in the subset of 26 acute COVID-19 donors for which sufficient cells were available. The results revealed a higher magnitude of CD4+ AIM reactivity with the CD4-RE pool compared to the S pool (*p* = 0.039 by Wilcoxon t test) in mild/moderate disease compared with severe disease (Figure 6A). These results confirm the previous acute setting observations in convalescent subjects [14,22] that responses in SARS-CoV-2 infection are multi-specific with recognition of viral antigens in addition to the S protein. 

When the ratio of CD4-RE to the S protein was calculated, the donors with mild/moderate disease had a significantly higher ratio than donors associated with severe disease (*p* = 0.024) (Figure 6B), indicating that more prominent recognition of non-S antigens is associated with milder disease. In conclusion, this data indicates that a polyantigenic T cell response is associated with mild/moderate disease as compared to severe disease. 

## 3. Discussion

In this study we analyzed T cell reactivity as a function of disease severity, in a cohort of early (<15 days PSO) versus late (up to 45 days PSO) acute COVID-19 patients. We also analyzed the breadth of responses and the relationships between CD4+ T cell responses and age and sex.

In terms of relationships between clinical outcomes and disease severity, previous studies have suggested an association between the magnitude of CD4 T cell responses in less severe disease [8,9,23,24], but these were limited by the number of acute COVID-19 subjects available for analysis (Mele et al., *n* = 55; Rydyznski Moderbacher et al., 2020 *n* = 24; Tan et al., 2021 *n* = 12; Oja et al., *n* = 56). Interestingly, Peng et al., reported broader and stronger T cell responses in convalescent subjects (at least 28 days PSO) who had severe disease. The authors speculate that this might be reflective of high viral loads associated with severe disease (and thus higher and more protracted stimulation) [25]. As such, these results are consistent with our observation that the correlation with mild disease is only observed in the early timepoints and lost thereafter. Our results are also consistent with a previous report by Oja et al., who performed similar experiments and reached identical conclusions in a smaller cohort of acute patients [24]. Our results confirm that CD4+ T cell responses in the acute phase of SARS-CoV-2 infection are associated with milder disease in a larger cohort of subjects (*n* = 89). 

We also extend these observations in terms of kinetics of responses. Our data highlight that the correlation of CD4+ T cell responses with milder COVID-19 is most prominent in the first 2 weeks of infection and becomes less prominent at later time points. Importantly, since these two studies were performed with samples from the first wave of COVID-19 disease obtained in North America (USA; Rydyznski Moderbacher et al., 2020) and Asia (Singapore, Tan et al., 2021), our study with samples from Italy, also derived from the first wave of COVID-19 disease, represents an important confirmation with comparable samples from a third continent and geographical location.

The Rydyznski Moderbacher et al., 2020 study also detected a less striking association between acute CD8+ responses and milder disease outcomes, which was not confirmed in our study, possibly because CD8+ responses were more difficult to detect, in part due to the higher background activation present in acute CD8+ T cells [26,27]. Relatively weak CD8+ responses in early acute donors were also reported by Zhou et al. [28]. When we compared our results with others at similar timepoints, we noted comparable frequencies of CD8+ T cell responses [8,29]; it is possible that these donors do develop CD8+ T cell responses at later timepoints. Finally, serum samples were not collected for all of the donors presented in this study, and thus correlations between humoral responses and disease severity were not addressed in the present study.

We also explored intrinsic characteristics of the donor cohort that could influence T cell reactivity. While males may have a higher risk of developing severe COVID-19 [17], we did not detect significant sex-based differences in acute CD4+ T cell responses, consistent with other reports [10,30]. However, we did see a correlation between age and the severity of acute COVID-19. This correlation has been observed in previous studies and is likely related to the smaller pool of naïve CD4+ T cells in older individuals [8,10]. 

A novel observation of the current study is that, beyond the correlation between CD4+ T cell responses and milder disease, a correlation exists with the breadth of response and disease severity, with prominent responses to non-S antigens being associated with milder disease outcomes. The data presented show that subjects mounting responses directed to additional targets beyond the spike protein are associated with milder disease. Based on the available data, it is not possible to further address whether that represents a broad response targeting many epitopes within the spike and non-spike pools, and/or an immunodominant response to a single antigen within the pools. The data also do not distinguish between a polyclonal response (broad T cell repertoire) and an oligoclonal response with just a few expanded clones. Additionally, the number of subjects for which enough cells were available to test non-spike peptides and for which cytokine profiles could be investigated is small, and, as such, no conclusions could be drawn regarding any functional differences (cytokine expression profile) between CD4+ T cells specific to the spike and CD4+ T cells specific to the non-spike.

These results are consistent with the report of Swadling et al., 2022, which highlighted that responses to NSP antigens were associated with favorable infection outcomes in a cohort of exposed health care workers [31]. These results imply that the ability to recognize multiple antigens is of potential relevance and should be considered in future vaccine designs [6,14].

## 4. Materials and Methods

### 4.1. Acute COVID-19 Donors

Donors in the acute phase of COVID-19 disease were enrolled at 3 sites in northern and central Italy: Genoa, Pavia, and Rome. At each location, donors gave informed consent and were enrolled in the study in compliance with the Helsinki Declaration as well as local ethical committees. In Genoa, 27 patients were enrolled at the Infectious Disease Unit of IRCCS Ospedale Policlinico San Martino. After blood was collected at the hospital, PBMCs were isolated from whole blood by researchers of the Biotherapy Unit in the Center of Excellence for Biomedical Research (CEBR) at the University of Genoa. In Pavia, blood was collected from 30 acute COVID-19 patients at the Fondazione IRCCS Policlinico San Matteo and PBMCs were processed by collaborators in the Division of Clinical Immunology and Infectious Diseases. In Rome, 32 COVID-19 patients donated blood at the University Hospital Policlinico Tor Vergata of Rome and PBMCs were isolated at the University of Rome Tor Vergata.

Each donor was confirmed positive for COVID-19 by either SARS-CoV-2 PCR or antibody (IgG or IgM) testing (see Table 1). Disease severity was classified as mild (*n* = 21), moderate (*n* = 35), or severe (*n* = 33) according to the World Health Organization (WHO) guidelines for the clinical management of COVID-19 (WHO/2019-nCoV/clinical/2020.5). In brief, donors with mild disease had mild clinical symptoms and no signs of viral pneumonia, while moderate disease was characterized by fever, cough, dyspnea, and symptoms indicating pneumonia. Severe disease was classified by symptoms of severe pneumonia and respiratory distress with a rate of > 30 breaths per minute. All acute COVID-19 donors were categorized by disease severity according to these guidelines. 

### 4.2. Isolation of Peripheral Blood Mononuclear Cells (PBMCs) 

At each site, whole blood was collected in ACD or heparinized tubes and centrifuged at 1850 rpm for 15 min to separate the plasma and cellular fractions. The plasma was carefully removed and stored at −20 °C. PBMCs were isolated by density gradient sedimentation with Ficoll-Paque (Lymphoprep, Nycomed Pharma, Oslo, Norway). The isolated PBMCs were cryopreserved by suspension in cell recovery media containing 10% DMSO (Gibco), supplemented with 10% heat-inactivated fetal bovine serum (FBS; Hyclone Laboratories, Logan UT) and stored in liquid nitrogen prior to shipment on dry ice to the La Jolla Institute for Immunology (San Diego, CA, USA) where the cells were again stored in liquid nitrogen until assayed. 

### 4.3. Peptide Synthesis and MP Preparation

All peptides used were synthesized as crude material (TC Peptide Lab, San Diego, CA, USA) and individually resuspended in dimethyl sulfoxide (DMSO) at a concentration of 10 or 20 mg/mL. For the SARS-CoV-2 S megapool, peptides were synthesized as 15-mers overlapping by 10 amino acids spanning the entire SARS-CoV-2 spike protein corresponding to the ancestral Wuhan sequence (GenBank: MN_908947). The SARS-CoV-2 CD4-RE megapool consists of experimentally defined epitopes from non-spike regions of the SARS-CoV-2 proteome and were selected based on recent meta-analysis (Grifoni et al., 2021). Peptides were synthesized and pooled to include both dominant and subdominant epitopes.

### 4.4. Flow Cytometry-Combined AIM + ICS T Cell Assay 

We have previously combined the activation-induced marker (AIM) assay and intracellular cytokine staining (ICS) assays as described in detail elsewhere (Tarke et al., Cell 2022). In the combined AIM + ICS assays in this study, PBMCs were cultured in the presence of SARS-CoV-2 S and CD4-RE-specific MPs (1 µg/mL) in 96-well U-bottom plates with 1 × 10^6^ PBMCs per well. As a negative control, an equimolar amount of DMSO was presented to the cells in triplicate wells. As a positive control, phytohemagglutinin (PHA, Roche, 1 µg/mL) was added to the cells in single wells. After incubation for 20 h at 37 °C, 5% CO_2_, Golgi-Plug containing brefeldin A, Golgi-Stop containing monensin (BD Biosciences, San Diego, CA, USA), and CD137 APC antibody were added to the 96-well plate. 

After an additional 4 h incubation, cells were stained on their membrane surface for 30 min at 4 °C in the dark with CD4 BUV395 (1:100; BD Biosciences Cat#563792), CD8 BUV496 (5:1000; Biolegend Cat#612942), CD14 BUV563 (1:100; BD Biosciences Cat#741360), CD19 BUV805 (1:100, BD Biosciences Cat#742007), Live/Dead eFluor 506 (1:1000; eBioscience Cat# 65-0866-18), CD69 BV605 (2:100; BD Biosciences 562989), CD3 AF532 (4:100, eBioscience Cat#58-0038-42), OX40 PE-Cy7 (25:1000; Biolegend Cat#350012), and CD137 APC (2:100; Biolegend Cat#309810). After washing, cells were then fixed with 4% paraformaldehyde in PBS (Sigma-Aldrich, St. Louis, MO, USA), permeabilized with saponin buffer (Sigma-Aldrich, St. Louis, MO, USA), and blocked for 15 min with saponin buffer supplemented with human serum (Gemini Bio-Products, Sacramento, CA, USA). Then the cells underwent a 30-min intracellular stain at room temperature with TNFɑ eFluor450 (1:100; Life Tech Cat#48-7349-42), IL-4 BV711 (1:100; BD Biosciences Cat#564112), IFNγ FITC (5:1000; Invitrogen Cat#11-73319-82), IL-2 BB700 (1:100; BD Biosciences Cat#566405), IL-17 PE (5:1000; Invitrogen Cat#12-7177-81), granzyme B AF700 (25:1000; BD Biosciences Cat#560213), and CD40L APC-efluor780 (1:100; eBioscience Cat#47-1548-42).

After washing, all samples were acquired on a Cytek Aurora flow cytometer (Cytek) and analyzed with FlowJo software (Tree Star). Gates for AIM or cytokine positive cells were drawn relative to the negative and positive controls for each donor (Figure S1). For both AIM and cytokine markers, the limit of sensitivity (LOS) was calculated by the median plus two-fold standard deviation of the background T cell reactivity in the DMSO control samples and the limit of detection (LOD) was calculated by the geometric mean plus the upper 95% confidence interval. 

### 4.5. Data Analysis and Statistics

All data analysis and statistics were performed with FlowJo 10 and GraphPad Prism 9.1. The figure legends contain all statistical details for the experiments. The geometric mean is indicated on each graph for data that are plotted in logarithmic scale. For non-logarithmic data, the median and 95% confidence intervals are shown. 

## Figures and Tables

**Table 1:** Description of acute COVID-19 donor cohort.

	Total (*n* = 89)	Mild (*n* = 21)	Moderate (*n* = 35)	Severe (*n* = 33)
**Collection Date**	February–May 2020	March–April 2020	March–May 2020	February–May 2020
SARS-CoV-2 PCR or Antibody positivity	100% (89/89)	100% (21/21)	100% (35/35)	100% (33/33)
PCR+	100% (85/85)	100% (18/18)	100% (35/35)	100% (32/32)
IgG+	96% (24/25)	100% (5/5)	100% (9/9)	91% (10/11)
IgM+	81% (22/27)	100% (11/11)	86% (6/7)	56% (5/9)
Days PSO				
Range	0–45,	0–33 ^#^	0–45	0–34 ^##^
(Median, IQR)	(10, 4)	(11, 4)	(11, 6)	(8, 3)
Gender				
Male	58% (52/89)	52% (11/21)	60% (21/35)	61% (20/33)
Female	42% (37/89)	47% (10/21)	40% (14/35)	39% (13/33)
Age (years)				
Range,	27–98	27–90	28–98	53–93 *
(Median, IQR)	(61, 49)	(48, 39)	(59, 47)	(75, 64)

^#^ 14% (3/21) not reported. ^##^ 3% (1/33) not reported. * 3% (1/33) not reported.

## Data Availability

The data presented in this study are available within this article or the supplementary materials.

## Supplementary Materials

The following supporting information can be downloaded at: https://www.mdpi.com/article/10.3390/ijms23137155/s1.
