# Supplementary material for: Early and Polyantigenic CD4 T Cell Responses Correlate with Mild Disease in Acute COVID-19 Donors
Source: Int J Mol Sci. 2022 Jun 28;23(13):7155. doi: 10.3390/ijms23137155 (PMC9267033; doi:10.3390/ijms23137155)
Supplement: Supplementary file 1 [file ijms-23-07155-s001.zip › Supplementary Figures v3/Figure S1.pdf]

Figure S1

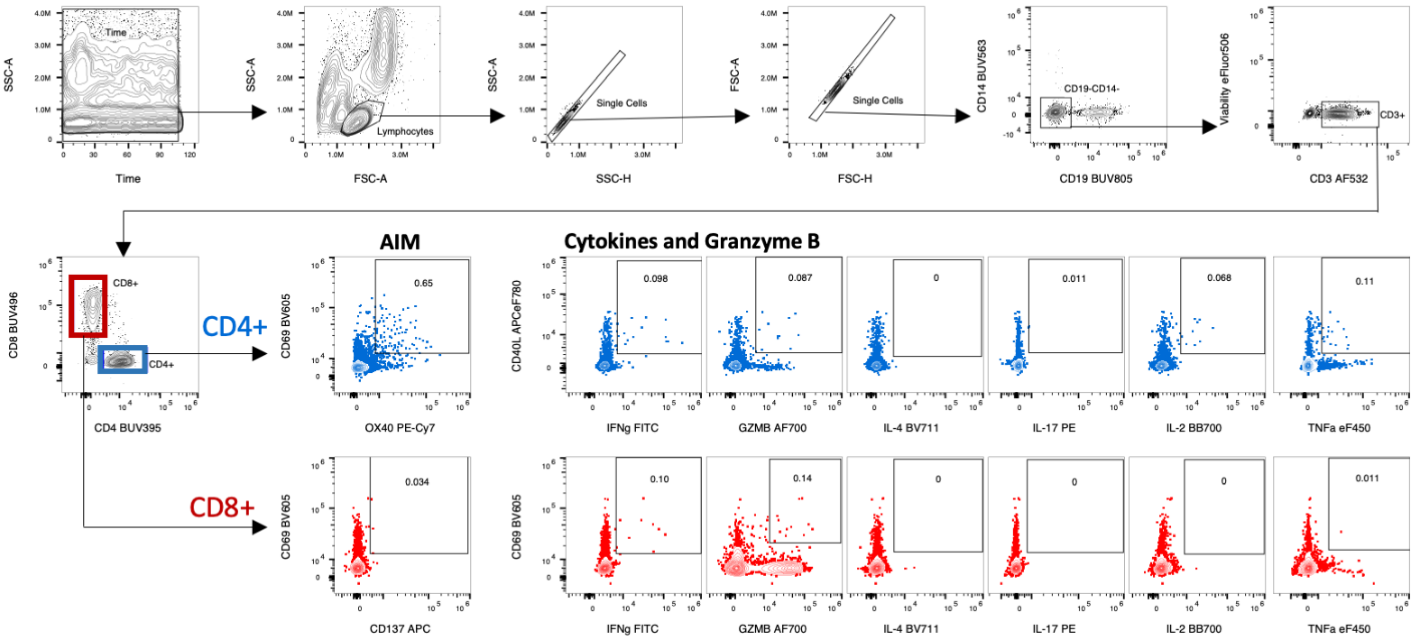

Figure S1. Gating strategy for AIM + ICS assay

The combined AIM + ICS assay was utilized for this study and a representative mild acute COVID-19 donor is shown for the gating strategy. In brief, the cells are sequentially gated for time, lymphocytes, singlets, and CD14-CD19-. Then CD3+ T cells are further gated on CD4+ or CD8+ T cells in order to define populations of AIM+, and cytokines or Granzyme B.
