# Supplementary material for: Early and Polyantigenic CD4 T Cell Responses Correlate with Mild Disease in Acute COVID-19 Donors
Source: Int J Mol Sci. 2022 Jun 28;23(13):7155. doi: 10.3390/ijms23137155 (PMC9267033; doi:10.3390/ijms23137155)
Supplement: Supplementary file 1 [file ijms-23-07155-s001.zip › Supplementary Figures v3/Figure S2.pdf]

## Figure S2

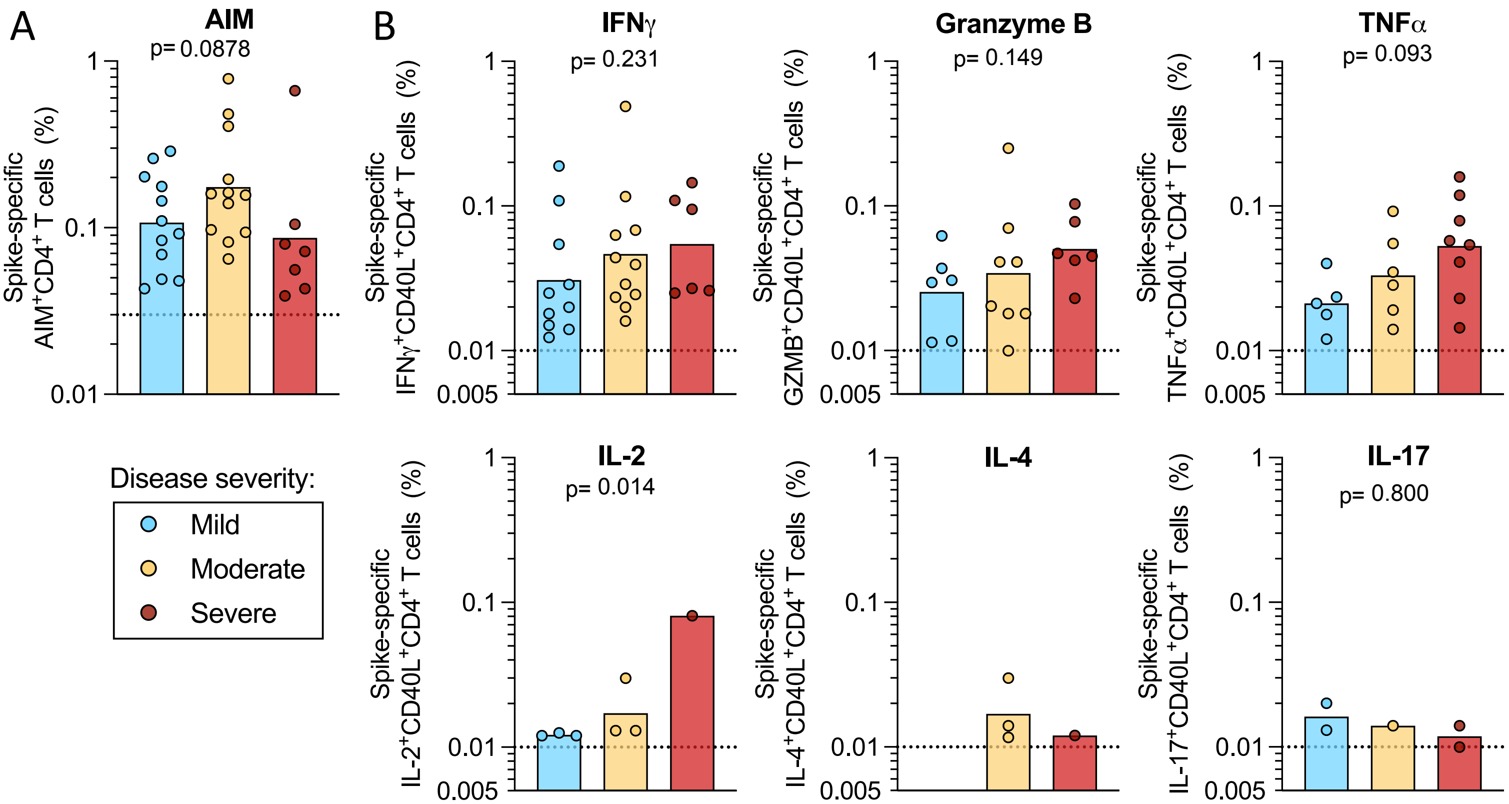


**Figure S2. CD4+ T cell responses to SARS-CoV-2 Spike in responding mild, moderate and severe COVID-19 donors**

PBMCs from acute COVID-19 donors were tested for CD4+ T cell responses to SARS-CoV-2 S by AIM + ICS assay. The donors tested were categorized as having mild (n=21), moderate (n=35), or severe (n=33) COVID-19 and only the donors with CD4+ T cell responses to S are plotted here. (**A**) CD4+ T cell responses to S as measured by the co-expression of OX40 and CD69. (**B**) CD4+ T cell responses were measured by the cells expressing CD40L in combination with production of IFN$\gamma$, Granzyme B, TNF$\alpha$, IL-2, IL-4 or IL-17. All data shown is background subtracted and SI>2. The geometric mean is represented by bars and the dotted line represents the LOS. The y-axis starts at the LOD. p values are calculated by Kruskal-Wallis One-way ANOVA and, when significant, further Mann-Whitney U tests are applied between disease severities and significant results are indicated by the symbols.
